# IL-4 Mediated Resistance of BALB/c Mice to Visceral Leishmaniasis Is Independent of IL-4Rα Signaling via T Cells

**DOI:** 10.3389/fimmu.2019.01957

**Published:** 2019-08-16

**Authors:** Emma McFarlane, Thabang Mokgethi, Paul M. Kaye, Ramona Hurdayal, Frank Brombacher, James Alexander, Katharine C. Carter

**Affiliations:** ^1^Strathclyde Institute of Pharmacy and Biomedical Sciences, University of Strathclyde, Glasgow, United Kingdom; ^2^Department of Biology, Centre for Immunology and Infection, Hull York Medical School, University of York, York, United Kingdom; ^3^International Centre for Genetic Engineering and Biotechnology, Cape Town Component, Cape Town, South Africa; ^4^Division of Immunology, Department of Pathology, Faculty of Health Sciences, Institute of Infectious Diseases and Molecular Medicine (IDM), South African Medical Research Council (SAMRC) on Immunology of Infectious Diseases, University of Cape Town, Cape Town, South Africa; ^5^Faculty of Health Sciences, Wellcome Centre for Infectious Diseases Research in Africa, Institute of Infectious Diseases and Molecular Medicine (IDM), University of Cape Town, Cape Town, South Africa; ^6^Department of Molecular and Cell Biology, University of Cape Town, Cape Town, South Africa

**Keywords:** *Leishmania donovani*, IL-4Rα, IL-4, T cell, mice

## Abstract

Previous studies infecting global IL-4Rα^−/−^, IL-4^−/−^, and IL-13^−/−^mice on a BALB/c background with the visceralizing parasite *Leishmania donovani* have shown that the T helper 2 cytokines, IL-4, and IL-13, play influential but not completely overlapping roles in controlling primary infection. Subsequently, using macrophage/neutrophil-specific IL-4Rα deficient BALB/c mice, we demonstrated that macrophage/neutrophil unresponsiveness to IL-4 and IL-13 did not have a detrimental effect during *L. donovani* infection. Here we expand on these findings and show that CD4^+^ T cell-(Lck^cre^), as well as pan T cell-(iLck^cre^) specific IL-4Rα deficient mice, on a BALB/c background, unlike global IL-4Rα deficient mice, are also not adversely affected in terms of resistance to primary infection with *L. donovani*. Our analysis suggested only a transient and tissue specific impact on disease course due to lack of IL-4Rα on T cells, limited to a reduced hepatic parasite burden at day 30 post-infection. Consequently, the protective role(s) demonstrated for IL-4 and IL-13 during *L. donovani* infection are mediated by IL-4Rα-responsive cell(s) other than macrophages, neutrophils and T cells.

## Introduction

Infection with the parasite *Leishmania donovani* causes visceral disease and can be fatal if it is not treated. Although there are major campaigns aimed at eliminating this disease e.g., World Health Organization 2020 roadmap, it still remains a serious neglected tropical disease (1) (https://www.who.int/leishmaniasis/en/), with no effective vaccine currently available (2). Successful pathogenesis is dependent on parasite survival in the host, a process mediated by a complex interplay of host factors. An in-depth understanding on the contribution of these factors to disease is therefore necessary to inform the development of novel or adjunct host-directed therapies (3, 4).

Earlier studies in this context revealed that the IFN-γ/IL-4 paradigm of resistance and susceptibility to intracellular infection, as defined for species causing cutaneous leishmaniasis (CL), does not apply holistically to species causing visceral leishmaniasis (VL). As with CL, protective immunity against this parasite relies on a Th1 response, which requires IL-12 production, and culminates in IFN-γ release (5, 6). In target tissues such as the liver, infection results in granuloma formation around infected macrophages (Kupffer cells) and eventual parasite death, primarily via the action of reactive nitrogen and oxygen intermediates (7, 8). However, unlike CL, a dominant inhibitory role for type 2 cytokines is less clear in murine models of VL (9). In asymptomatic and cured VL patients (10–12), both IFN-γ and IL-4-producing T cells have been identified and in the murine model of VL, protection is related to higher frequencies of cytokine-producing cells rather than altering the IFN-γ/IL-4 balance (13). In contrast, both human (12, 13) and murine (14) VL studies show that IL-10 is more important than IL-4 in immune suppression and parasite persistence.

Rather than being a detrimental cytokine for host protection, the evidence tends to suggest that type 2 immune responses may actually contribute to control of VL. Accordingly, our previous studies utilizing gene-deficient mice have identified protective roles for IL-4, IL-13, and IL-4Rα signaling during primary *L. donovani* infection (15–17). Control of parasite growth within the liver depends on the ability of Kupffer cells to clear parasites inside mature granulomas (15), a mechanism which requires T cell-derived IFN-γ (18) and the coordinated activity of macrophages which migrate toward the infected area. Enhanced susceptibility of IL-4^−/−^, IL-13^−/−^, and IL-4Rα^−/−^ mice to *L. donovani* infection was associated with a reduction in type 1 responses and retarded granuloma maturation so that fewer mature or sterile granulomas were present (15, 16, 19). In line with these observations, a recent study indicated that IL-10, and not IL-4, was responsible for manipulating monocytes/macrophages in VL infection (20). In addition to playing significant roles in controlling primary infection with *L. donovani*, IL-4, and IL-13 have also been associated with the successful outcome of sodium stibogluconate (SSG) treatment and vaccination with recombinant hydrophilic acylated surface protein (HASP)B-1 (15, 17, 19). While these studies added value to our understanding of the contribution of IL-4/IL-13 in host-protection against VL, they did not provide information on which cells were targeted by these cytokines via the IL-4Rα, and are therefore critical for protective immunity. Indeed, both IL-4 and IL-13 are pleiotropic cytokines and numerous cell types of both the innate and adaptive immune system produce and respond to these cytokines (21). In this regard, studies in cell-type specific IL-4Rα-deficient mice during CL has revealed a hierarchical interaction between the IL-4Rα chain and its ligands on different immune cells. To illustrate this, IL-4Rα signaling via DCs to produce IL-12 plays a protective role during cutaneous infection with *Leishmania major* (22), while IL-4Rα signaling via T cells (23) and Th2 induction, via macrophages and alternative activation (24), and via B cells and IL-4 production (25), all promote disease progression.

To further dissect the cell-specific requirements of IL-4/IL-13 signals on immune cells in VL, we used conditional cell-specific IL-4Rα deficient BALB/c mice, generated by the cre/*loxP* recombination system, to demonstrate that macrophage/neutrophil-specific (LysM) IL-4Rα signaling was not necessary for an effective healing response during VL, nor did it influence the outcome of SSG chemotherapy (16). Other possible target cells for IL-4 during VL may include dendritic cells (DC) (26, 27) and B cells (28) but more particularly CD4^+^ (26, 29) and/or CD8^+^ (30) T cells, whose active involvement has been shown not only to be essential to control primary infection and granuloma formation but also for successful SSG chemotherapy and therapeutic vaccination (15, 31, 32). Consequently, in this study we generated CD4^+^ T cell-specific IL-4Rα^−/−^ (Lck^cre^IL-4Rα^−/lox^) mice (23) and iLck^cre^IL-4Rα^−/lox^ mice that lack IL-4Rα on both CD4 and CD8 T cells (33) to determine the temporal role of IL-4 signaling via CD4^+^ and CD8^+^ T cells on the progression of VL infection. Unlike global IL-4Rα^−/−^ mice infected with *L. donovani* that developed significantly higher parasite burdens than wild-type mice in this and previous studies (15), CD4^+^ T cell specific IL-4Rα^−/−^ mice were by comparison resistant to infection. Indeed, at day 30 post-infection CD4^+^ T cell as well as pan T cell-specific IL-4Rα^−/lox^ mice (iLck^cre^IL-4Rα^−/lox^) were more^.^ resistant than their wild-type littermate controls to hepatic infection with *L. donovani*. Increased susceptibility in global IL-4Rα^−/−^ mice was associated with a diminished type 1 response and increased IL-10 production while CD4^+^ T cell deficient IL-4Rα^−/lox^ mice had comparable expression of IFN-γ on both CD4^+^ and CD8^+^ T cells and serum IL-10 levels similar to infected wild-type mice. Consequently, the protective effects of IL-4 during primary *L. donovani* infection are not mediated via direct effects on either CD4^+^ or CD8^+^ T cells, and IL-4 may even play a small regulatory role in these cells.

## Materials and Methods

### Ethics Statement

Animal experiments and experimental procedures were carried out in line with UK Home Office regulations and the University of Strathclyde Animal Welfare and Ethical Review Board regulations under project license number: PPL 60/3525. BALB/c mice were all bred and maintained in the Biological Procedures Unit at the University of Strathclyde, Glasgow and experimental design and reporting adhere to the ARRIVE guidelines.

### Animals and Parasites

Lck^cre^IL-4Rα^−/lox^ BALB/c mice were created as described (23). Briefly, Lck^cre^IL-4Rα^−/lox^ mice were generated by inter-crossing IL-4Rα^lox/lox^ BALB/c mice (34) with IL-4Rα^−/−^ BALB/c mice (35) and BALB/c mice expressing the Cre-recombinase under the control of *Lck* locus, a T cell specific promoter (36) to generate hemizygous Lck^cre^IL-4Rα^−/lox^ mice. The efficiency of the deletion was characterized (23) whereby Lck^cre^IL-4Rα^−/lox^ BALB/c mice have IL-4Rα selectively deleted from CD4^+^ T cells (CD4^+^ T cell specific IL-4Rα deficient mice). As these mice have variable and incomplete deletion in CD8^+^ T cells (28), mice lacking the IL-4Rα on all T cells (iLck^cre^IL-4Rα^−/lox^ mice) were produced as described (33). The iLck^cre^ construct was designed with a nuclear localization signal and an eukaryotic translation start site at the 5' end of the *Cre*-recombinase, which meant that the insert integrated downstream from the *Lck* proximal promoter (33, 37). Non-T cell populations such as B cells, macrophages and DC express IL-4Rα as normal. PCR genotyping studies were used to identify Lck^cre^IL-4Rα^−/lox^ mice. Lck^cre^-negative IL-4Rα^−/lox^ littermate BALB/c mice, which express similar levels of IL-4Rα as wild-type BALB/c mice, were used as controls to iLck^cre^IL-4Rα^−/lox^ mice, herein referred to as wild-type (WT) littermate control.

Commercially obtained Golden Syrian hamsters (*Mesocricetus auratus*) were used for maintenance of *L. donovani* (Harlan Olac, Bicester, UK) and *L. donovani* strain MHOM/ET/67:LV82 was used in these studies (38). Mice, age and sex-matched, were coded and randomized before injection to avoid bias. The mice were infected (day 0) by intravenous injection into the tail vein without using an anesthetic with 1–2 × 10^7^ *L. donovani* amastigotes (16). Depending on the experiment, mice were killed (*n* = 4/5 per group) at days 14 or 15, 30, and 56 or 60 post-infection (p.i.).

### *In vivo* Studies

Liver, spleen, and bone marrow impression smears from each mouse were prepared on a glass microscope slide at sacrifice. The slides were fixed in methanol for 2 min, stained with a 10% Giemsa solution (BDH, VWR International Ltd, UK) for 20 min, washed in tap water and allowed to dry. The number of parasites /1,000 host nuclei for each sample was determined at ×1,000 magnification. Blood was collected from each mouse at sacrifice and left to clot. The resulting serum was harvested, stored at −20°C, and then used in assays to determine specific antibody titers and cytokine levels.

### Spleen Cell Proliferation Assays

Mouse spleens were removed aseptically at sacrifice, and processed as described previously (16). Splenocytes, seeded at 5 × 10^5^ cells/well, were incubated at 37°C and 5% CO_2_/95% air for 72 h with medium alone (unstimulated controls), 25 μg/ml *L. donovani* promastigote soluble antigen (SAG) or 10 μg/ml ConA (Sigma-Aldrich, Poole, UK). The plates were then stored at −20°C and cell supernatants were used in cytokine/nitrite assays.

### Cytokine Production

Enzyme-linked immunosorbent assays (ELISA) was used to determine IFN-γ levels in splenocytes supernatants from lymphocyte proliferation assays and serum samples using the method described (19). A volume of 50 μl/well of 1 μg/ml w/v of the appropriate purified anti-mouse capture antibody [IL-10 JES5-2AS and IFNγ R4-6A2 (PharMingen, supplied by Insight Biotechnology, Wembley, UK) or IL-13 38213 (R&D Systems Europe Ltd, Abingdon, UK)] and 100 μl/well of the appropriate biotinylated rat anti-mouse monoclonal antibody at 2 μg/ml [IL-10 SXC-1 and IFNγ XMG1-2 (PharMingen, supplied by Insight Biotechnology, Wembley, UK) or IL-13 (R&D Systems Europe Ltd, Abingdon, UK)] was used in assays. The absorbance of the samples was read at 405 nm using a SOFTmax Pro (Molecular devices, California, USA) and cytokine concentrations (ng/ml) in samples was determined by linear regression using the standard values. In all cases the correlation coefficient was 0.970 or better.

### Specific Antibody Titers

Specific IgG1 and IgG2a titers were determined for serum samples using the method described previously (16). Plates were coated with 100 μl/well of 10 μg/ml *L. donovani* SAG and probed using anti-mouse IgG1 (1:20,00 dilution) or IgG2a (1:10,000 dilution) HRP conjugates (Southern Biotechnology, supplied by Cambridge BioScience Ltd, Cambridge, UK). The absorbance of samples was determined at 450 nm using a SOFTmax Pro (Molecular devices, California, USA) and end point titers, defined as the last dilution to give an absorbance above background levels, was determined.

### Determination of IgE Production

A 96 well microtiter plate (Greiner Bio-One, Germany) was coated with 50 μl/well of 1 μg/ml IgE purified anti-mouse capture antibody (clone R35-72 obtained from PharMingen and supplied by Insight Biotechnology, Wembley, UK) diluted in PBS pH 9.0 and incubated at 4 °C overnight. The plate was washed three times with PBS supplemented with 0.05% v/v Tween 20 (Sigma-Aldrich, Poole, UK), dried and blocked with 10% v/v FCS/PBS. Following addition of 200 μl/well the plate was incubated at 37°C for 1 h. The plate was washed 3 times, dried and serum samples diluted at 1/50 were serially diluted in 10% v/v FCS/PBS along the plate. The plate was incubated at 37°C for 2 h. Following incubation, the plate was washed 4 times and 100 μl/well of a biotinylated rat anti-mouse IgE monoclonal antibody (clone R35-118 obtained from PharMingen and supplied by Insight Biotechnology, Wembley, UK) diluted 1/5,000 in 10% v/v FCS/PBS was added to the plate. The plate was then incubated at 37°C for 1 h. The plate was washed 5 times and 100 μl/well of streptavidin alkaline phosphatase (obtained from PharMingen and supplied by Insight Biotechnology, Wembley, UK) diluted at 1/2,000 in 10% v/v FCS/PBS was added. The plate was incubated at 37°C for 45 min. The plate was washed 6 times and 100 μl/well of substrate (1 mg/ml p-nitrophenylphosphate (Sigma-Aldrich, Poole, UK) in 0.1 M glycine buffer, pH 10.4) was added. The plate was incubated in the dark at 37°C until an appropriate yellow color developed. The absorbance of the samples at 405 nm was determined using a SOFTmax Pro (Molecular devices, California, USA) and the endpoint dilution determined as described above.

### Histology

Sections of liver were removed at sacrifice, fixed in neutral buffered formalin, and then processed for staining with Haematoxylin and Eosin (Fisher Scientific, Loughborough, UK). Granulomas were scored on their level of maturity based on the following criteria; infected Kupffer cells (parasitized macrophages), immature (developing granuloma consisting of CD4^+^ and CD8^+^ T cells and monocytes surrounding infected Kupffer cells), mature (more developed than immature) or sterile (parasite free granuloma) (39).

### Flow Cytometry

The percentage of CD4^+^IFNγ^+^ and CD8^+^IFNγ^+^ cells present in the spleen of mice was determined by flow cytometry. Single cell suspensions were prepared from the spleens of uninfected and infected mice in complete medium [RPMI 1640 (Lonza, Belgium) supplemented with 1% v/v of 2 mM L-glutamine solution and 1% v/v of 100 IU/ml Penicillin-100 μg/ml Streptomycin (PAA Laboratories, GmbH, Austria), and 10% v/v FCS (Sigma Aldrich, Poole, UK)]. Cell suspensions were pelleted by centrifugation at 1,000 rpm, 4°C for 5 min and the pellets were resuspended in 3 ml of erythrocyte lysing solution (0.007 M ammonium chloride, 0.085 M Tris, pH 7.2). Cell suspensions were incubated at 37°C for 5 min, washed three times in complete medium, and then resuspended at a concentration of 1 × 10^6^/ml. The cells were incubated for 4 h with 2 μl/ml Brefeldin A (BD Biosciences, UK), 500 ng/ml ionomycin and 50 ng/ml phorbol 12-mysristate 13 acetate (Sigma Aldrich, Poole, UK) at 37°C and 5% CO_2_. The cells were then washed with FACS buffer (5% w/v Bovine serum albumin, Fraction V (Sigma Aldrich, Poole, UK), 2 mM EDTA in PBS pH 7.4) for 5 min at 500 g, and then resuspended in FACS buffer containing 1 μg/ml purified rat anti-mouse CD16/CD32 (FCγ111/11 receptor) monoclonal antibody (BD Biosciences, Oxford, UK) and incubated for 20 min. The cells were washed as before and stained with the appropriate anti-mouse antibody diluted in FACS buffer (0.5 μg/ml PerCP-labeled anti-CD4^+^ or 1 μg/ml APC-labeled anti-CD8^+^ or 0.5 μg/ml PerCP and 1 μg/ml APC-labeled IgG isotype controls, BD Biosciences, Oxford, UK) in the dark for 60 min at 4°C. Cells were washed three times and then incubated with a 10% v/v cell lysing solution diluted in distilled water (BD Biosciences, Oxford, UK) for 10 min in the dark. The cells were washed again and incubated for a further 10 min in the dark with a 10% v/v permeabilizing solution diluted in distilled water (BD Biosciences, Oxford UK). Following this incubation, cells were washed and resuspended in 2 μg/ml purified rat anti-mouse CD16/CD32 (FCγ111/11 receptor) monoclonal antibody (BD Biosciences, Oxford, UK) diluted in FACS buffer. After a 10 min incubation, PE-labeled anti-mouse IFNγ (final concentration 2 μg/ml) and PE-labeled IgG isotype control (final concentration 2 μg/ml, BD Biosciences, Oxford, UK) diluted in 10% v/v permeabilizing solution was added to the appropriate sample and the cells were incubated for 60 min. The cells were washed once more, and then resuspended in 400 μl of a 10% v/v cell fix solution diluted in distilled water (BD Biosciences, Oxford, UK). The number of positive staining cells for a specific marker was determined using a FACSCanto^TM^ (BD Biosciences, Oxford, UK). Color compensation using BD^TM^ Compbeads and the antibodies used to stain cells was carried out before cell data was collected based on forward and side scatter using FACsDiva^TM^ software.

### Statistical Analysis of Data

Downstream data analysis was performed blind to treatment group and experiments were repeated at least twice when significant differences between treatments were obtained. Parasite burden from *in vivo* experiments were analyzed using a one-way ANOVA using log_10_ transformed data, followed by a Fisher's PLSD test to analyze differences between treatments using Statview^®^ version 5.0.1 software package. Significant differences between treatments for cytokine and flow cytometry data were identified using a Kruskal Wallis test followed by a Tukey test. Granuloma maturation data was analyzed using a χ^2^ test and the mean % granulomas in the Kupffer cell, immature, mature and sterile categories. Data was analyzed using Minitab Express^TM^ version 1.51 software package and a *p* < 0.05 was considered significant.

## ResultS

### A Protective Role for IL-4 During *L. donovani* Infection Involves Cell Targets Other Than CD4^+^ T Cells

CD4^+^ T cell-specific IL-4Rα deficient (Lck^cre^IL-4Rα^−/lox^) and wild-type littermate control (IL-4Rα^−/lox^) and global IL-4Rα^−/−^ BALB/c mice were infected with *L. donovani* on day 0 and parasite burdens in the spleen, liver, and bone marrow were determined, on day 14/15, 30, and 56/60 p.i. All three mouse strains had similar parasite burdens in the spleen, liver, and bone marrow at early times p.i. (day 14 or 15; Table S1), indicating that the inability to signal through IL-4Rα on CD4^+^ T cells did not interfere with establishment and early control of infection in the spleen, liver, and bone marrow.

**Figure 1:**
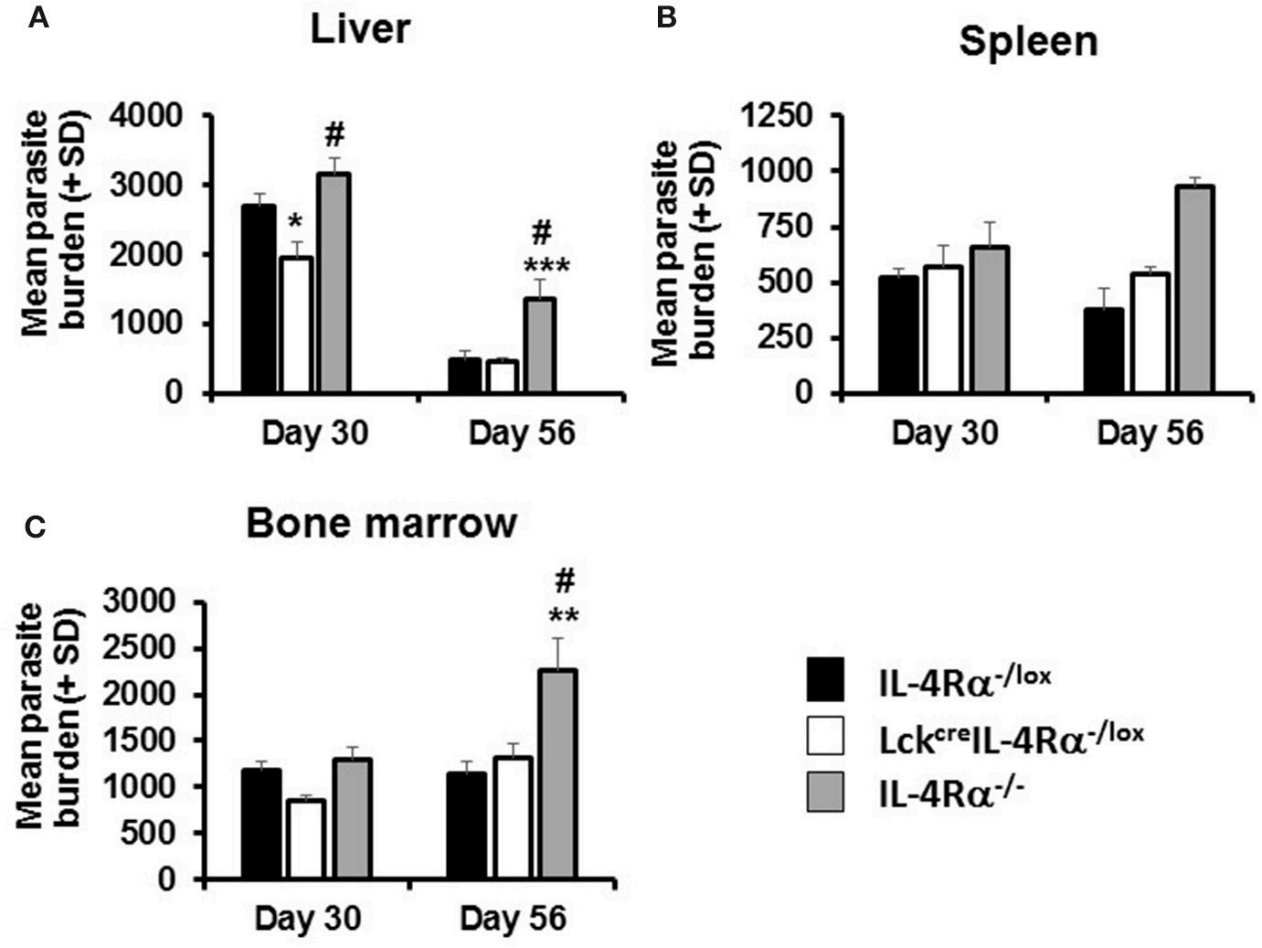
Parasite burdens in wild-type (IL-4Rα^−/lox^), CD4 T cell-specific IL-4Rα deficient (Lck^cre^IL-4Rα^−/lox^), and IL-4Rα^−/−^ BALB/c mice were determined at days 30 and 56 post-infection with 1–2 × 10^7^ *L. donovani* amastigotes in the liver **(A)**, spleen **(B)**, and bone marrow **(C)** by LDU. Representative data from one of three experiments performed (*n* = 5/group). ^*^p < 0.05, ^**^p < 0.01, ^***^p < 0.001 compared to WT control (IL-4Rα^−/lox^), ^#^*p* < 0.05 Lck^cre^IL-4Rα^−/lox^ compared to IL-4Rα^−/−^.

At day 30 p.i., CD4^+^ T cell IL-4Rα deficient mice and littermate controls had comparable parasite burdens in the spleen and bone marrow. However, CD4^+^ T cell IL-4Rα deficient mice had significantly lower liver parasite burdens than WT littermate control and global IL-4Rα^−/−^ BALB/c mice (Figure 1) at this time. This effect was transient however, and by day 56 parasite burdens in all three sites was comparable between WT and CD4^+^ T cell-specific IL-4Rα deficient mice. In contrast, and as expected (15), global IL-4Rα^−/−^ mice were more susceptible to *L. donovani* infection and this was exhibited by significantly higher parasite burden at later time points in all target tissues (Figure 1). Thus, selective deficiency of IL-4Rα expression in CD4^+^ T cells had a temporary protective effect, which was only expressed in the liver, but did not alter the overall susceptibility to VL.

### IL-4 Signaling Via CD4^+^ T Cells Is Not a Requirement for Effective Hepatic Granuloma Formation Following *L. donovani* Infection

**Figure 2:**
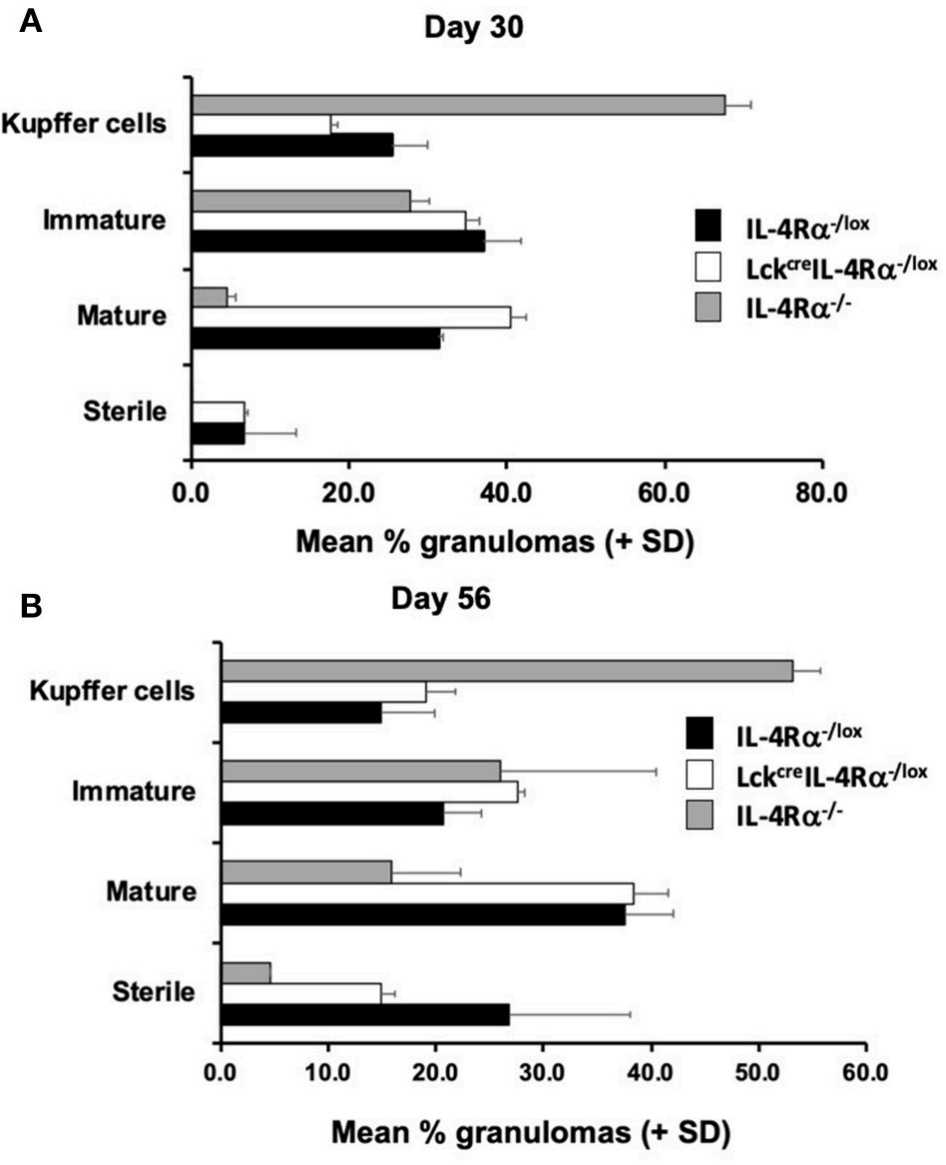
CD4^+^ T cell specific IL-4Rα^−/lox^ (Lck^cre^IL-4Rα^−/lox^), wild-type control (IL-4Rα^−/lox^), and global IL-4Rα^−/−^ BALB/c mice were infected with *L. donovani* on day 0 post-infection and sacrificed at days 30 **(A)** and 56 **(B)** post-infection. At each time-point, sections of liver were removed, processed, and stained with haematoxylin and eosin to enable scoring of hepatic liver granulomas. Representative data from one of two experiments performed (*n* = 5/group). On day 30 **(A)** the distribution was significantly different for control and global IL-4Rα^−/−^ mice (χ^2^ = 44; *p* < 0.00001) and CD4^+^ T cell specific IL-4Rα^−/lox^ (Lck^cre^IL-4Rα^−/lox^) and global IL-4Rα^−/−^ mice (χ^2^ = 63; *p* < 0.00001). On day 56 **(B)** the distribution was significantly different for control and global IL-4Rα^−/−^ mice (χ^2^ = 46; *p* < 0.00001) and CD4^+^ T cell specific IL-4Rα^−/lox^ (Lck^cre^IL-4Rα^−/lox^) and global IL-4Rα^−/−^ mice (χ^2^ = 30; *p* < 0.00001).

**Figure 3:**
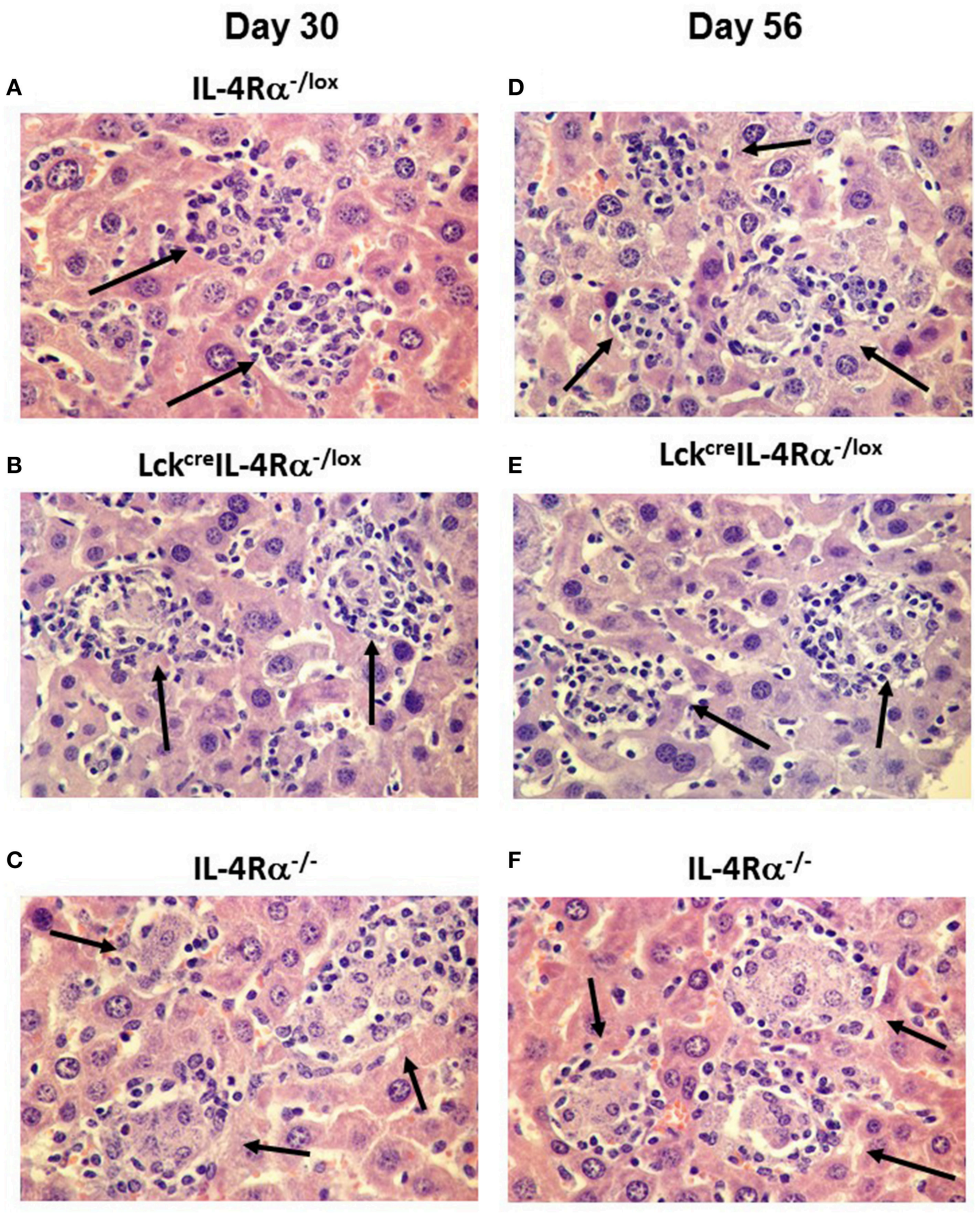
Representative photomicrographs of the hepatic granuloma response at days 30 and 56 in *L. donovani* infected wild-type (IL-4Rα^−/lox^) **(A,D)**, CD4^+^ T cell specific IL-4Rα^−/lox^ (Lck^cre^IL-4Rα^−/lox^) **(B,E)** and global IL-4Rα^−/−^ mice **(C,F)**. Mature granulomas (arrows) in IL-4Rα^−/lox^ mice and Lck^cre^IL-4Rα^−/lox^ mice are shown in day 30 whilst global IL-4Rα^−/−^ mice show abrogated granuloma development characterized by immature granulomas and amastigotes within the cytoplasm of infected Kupffer cells (arrows). At day 56 post-infection, IL-4Rα^−/lox^ mice and Lck^cre^IL-4Rα^−/lox^ mice show sterile granulomas (arrows) whilst heavily parasitized immature granulomas remain in the livers of global IL-4Rα^−/−^ mice (arrows). Magnification 400×.

Granuloma formation in livers of *L. donovani* infected mice was assessed at days 15, 30, and 56 post-infection in CD4^+^ T cell specific IL-4Rα deficient (Lck^cre^IL-4Rα^−/lox^), wild-type littermate control (IL-4Rα^−/lox^) and global IL-4Rα^−/−^ BALB/c mice. At day 15 post-infection, although granuloma development was evident, the granulomas were at an immature stage and, as such, no significant differences between groups were observed (Table S2). At day 30 post-infection, however, CD4^+^ T cell-specific IL-4Rα deficient mice and wild-type controls showed evidence of granuloma maturation, as defined by an increased number of mature and sterile granulomas, compared with global IL-4Rα^−/−^ mice (Figure 2A). Similarly, at day 56, the frequency of mature and sterile granulomas had increased and was similar in CD4^+^ T cell-specific IL-4Rα deficient mice and wild-type control mice, reflecting a similar ability to control liver parasite burdens by this time point (Figure 2B). Granuloma maturation was significantly retarded in global IL-4Rα^−/−^ but not CD4 T cell-specific compared with WT mice at both time points (*p* < 0.00001). Representative photomicrographs show granuloma formation in each group at days 15 (Figure S1), 30 (Figures 3A–C) and day 56 (Figures 3D–F) post-infection.

### Th2/type 2 Immune Responses Are Unchanged in CD4 T Cell-Specific IL-4Ra Deficient Mice in Response to *L. donovani*-induced VL

**Figure 4:**
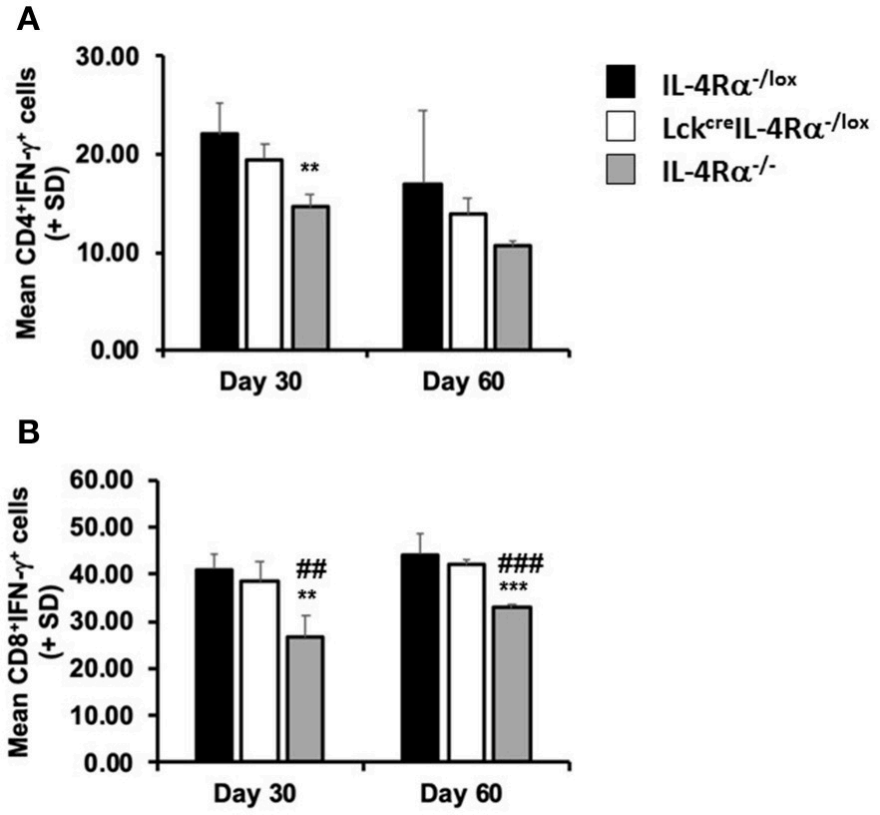
CD4^+^ T cell specific IL-4Rα deficient (Lck^cre^IL-4Rα^−/lox^), wild-type control (IL-4Rα^−/lox^) and global IL-4Rα^−/−^ mice were sacrificed at days 30 and 60 post-infection. At each time-point, splenocytes were removed, stimulated with ionomycin and PMA in the presence of brefeldin A, and the percentage of IFN-γ secreting CD4^+^ **(A)** and CD8^+^ **(B)** cells were measured by flow cytometry. Representative data from one of two experiments performed. ^**^p < 0.01, ^***^p < 0.001 compared to WT control (IL-4Rα^−/lox^), ^##^*p* < 0.01, ###*p* < 0.001 Lck^cre^IL-4Rα^−/lox^ compared to IL-4Rα^−/−^.

An inability of global IL-4Rα^−/−^ mice to control parasite burdens or develop a mature and effective granulomatous response has previously been associated with a down-regulation in serum IFN-γ production (15). In the present study we therefore measured production of IFN-γ by antigen-stimulated spleen cells and the frequency of CD4^+^IFN-γ^+^ and CD8^+^IFN-γ^+^ splenocytes of each mouse group after stimulation with PMA and ionomycin, as a measure of commitment to cytokine production during infection. On day 30 post-infection, we found no significant difference in the amount of IFN-γ [ng/ml] produced by antigen-stimulated splenocytes from any group of mice, WT controls (0.207 ± 0.030), CD4^+^ T cell specific IL-4Rα deficient mice (0.331 ± 0.049), global IL-4Rα^−/−^ mice (0.482 ± 0.076). However, using flow cytometry to examine intracellular cytokine production at this time revealed that the percentages of CD4^+^IFN-γ^+^ splenocytes (Figure 4A) and CD8^+^/IFN-γ^+^ splenocytes (Figure 4B) in global IL-4Rα^−/−^ mice were significantly lower (CD4^+^IFN-γ^+^, *p* < 0.05; CD8^+^IFN-γ^+^, *p* < 0.02) than observed in WT controls while the frequency of CD8^+^/IFN-γ^+^ splenocytes in global IL-4Rα^−/−^ mice was also reduced compared with CD4^+^ T cell specific IL-4Rα^−/−^ mice (*p* < 0.01, Figure 4B). This pattern was repeated on day 60 post-infection, (e.g., *p* < 0.0001 for global IL-4Rα^−/−^ mice vs. CD4^+^ T cell-specific IL-4Rα-deficient mice). There was no significant difference measured in the frequency of CD4^+^IFN-γ^+^ and CD8^+^IFN-γ^+^ cells between CD4^+^ T cell specific IL-4Rα deficient mice and wild-type controls at all time points. Together, these data indicate that IFN-γ-responses in Lck^cre^IL-4Rα^−/lox^ mice developed fully in the absence of IL-4Rα^+^ signaling on CD4^+^ T cells when compared with wild-type mice over the course of infection. Moreover, enhanced susceptibility to *L. donovani* infection in global IL-4Rα^−/−^ mice is associated with reduced IFN-γ secretion by CD4^+^ and CD8^+^ T cells.

**Figure 5:**
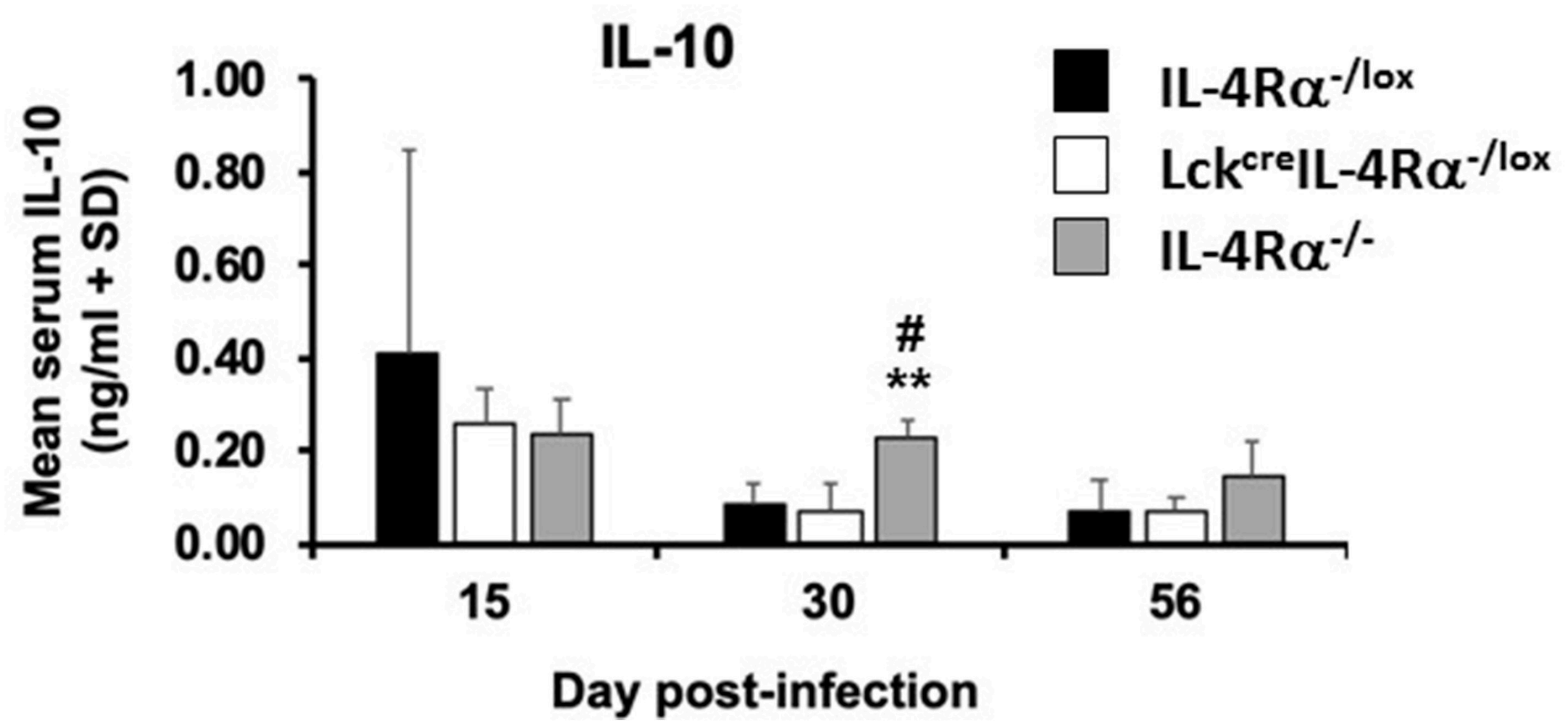
Serum IL-10 levels in wild-type control mice (IL-4Rα^−/lox^), CD4^+^ T cell specific IL-4Rα deficient mice (Lck^cre^IL-4Rα^−/lox^) and global IL-4Rα^−/−^ over the course of infection were determined by ELISA against *Leishmania donovani* soluble antigen. Representative data from one of two experiments performed (*n* = 5/group). ^**^p < 0.01 compared to WT control (IL-4Rα^−/lox^), ^#^*p* < 0.05 Lck^cre^IL-4Rα^−/lox^ compared to IL-4Rα^−/−^.

IL-10 levels are associated with susceptibility to *L. donovani* infection (14), thus, IL-10 levels were measured in cell supernatants of antigen-stimulated spleen cells and in the serum from CD4^+^ T cell specific IL-4Rα deficient mice, wild-type control mice and global IL-4Rα^−/−^ mice at days 15, 30, and 56 post-infection. There was no significant difference in the amount of IL-10 [ng/ml] produced by antigen-stimulated cells from any group of mice at day 30, WT controls (0.281 ± 0.075), CD4^+^ T cell specific IL-4Rα deficient mice (0.231 ± 0.026), and global IL-4Rα^−/−^ mice (0.230 ± 0.013). In addition, while similar levels of IL-10 were present in the serum of all three groups of mice at day 15 p.i, by day 30 p.i significantly lower concentrations of IL-10 were present in the serum of wild-type control (*p* < 0.01) and CD4^+^ T cell specific IL-4Rα deficient mice (*p* < 0.01) compared with global IL-4Rα^−/−^ mice (Figure 5). This pattern was repeated on day 56 post-infection. This data suggests that the relative resistance observed in CD4^+^ T cell specific IL-4Rα deficient and littermate control mice, in comparison to highly susceptible global IL-4Rα^−/−^ mice, is associated with comparatively limited IL-10 production.

### CD4^+^ T Cell Specific IL-4Rα-deficient Mice Develop *L. donovani*-specific IgG1 and IgE Antibody Responses Similar to WT Control Mice

**Figure 6:**
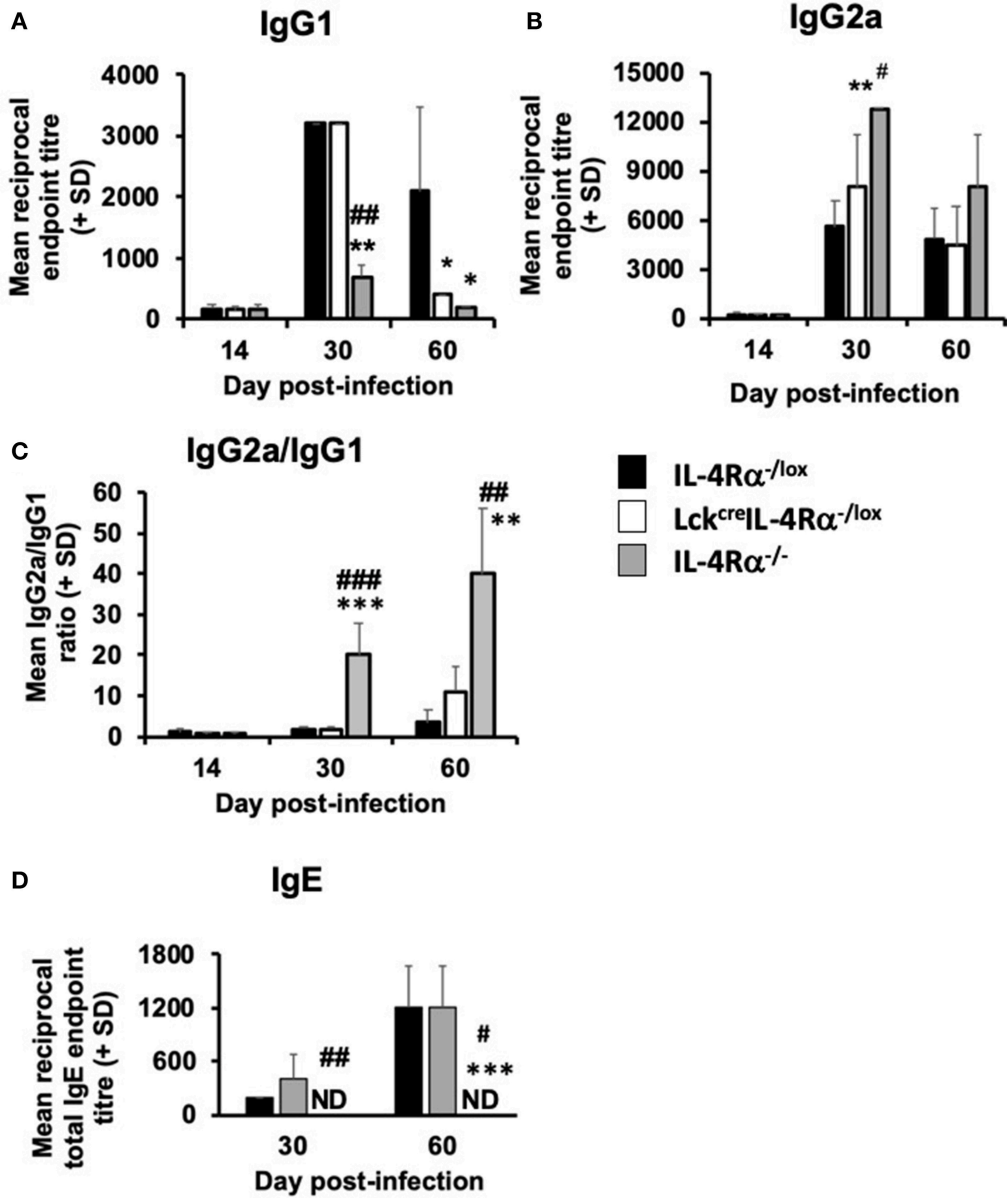
The effect of *L. donovani* infection on the antibody response of *L. donovani* infected CD4^+^ T cell specific IL-4Rα deficient (Lck^cre^IL-4Rα^−/lox^), wild-type control (IL-4Rα^−/lox^), and global IL-4Rα^−/−^ mice. Antigen-specific IgG1 **(A)**, IgG2a **(B)**, the ratio of antigen-specific IgG2a/IgG1 **(C)** and total IgE serum levels are shown **(D)**. Specific antibody titers were determined using an antigen-specific ELISA and total IgE levels were also determined using a direct ELISA assay. ^*^p < 0.05, ^**^p < 0.01, ^***^p < 0.001 compared to WT control (IL-4Rα^−/lox^), #*p* < 0.01, ##*p* < 0.01, ###*p* < 0.001 Lck^cre^IL-4Rα^−/lox^ compared to IL-4Rα^−/−^. Representative data from one of two experiments performed (*n* = 5/group).

*L. donovani*-specific IgG1 and IgG2a levels and total serum IgE levels were measured in CD4^+^ T cell specific IL-4Rα^−/−^ mice, wild-type control and global IL-4Rα^−/−^ mice at days 15, 30, and 56 post-infection. There was no difference in specific IgG1 or IgG2a in the three groups of mice at day 14 post-infection (Figures 6A,B). However at day 30 p.i., global IL-4Rα^−/−^ produced significantly lower levels (*p* < 0.0.01) of antigen-specific IgG1 but significantly higher IgG2a levels compared with CD4^+^ T cell specific IL-4Rα deficient mice and wild-type controls. In contrast, IgG1 and IgG2a titers were similar in CD4^+^ T cell specific IL-4Rα deficient mice and wild-type controls (Figures 6A,B). On day 60 p.i., both global and CD4^+^ T cell specific IL-4Rα^−^deficient mice had significantly lower IgG1 titers compared with WT controls (*p* < 0.05). IgG2a titers were similar in all three groups of mice (Figures 6A,B). Comparison of the ratio of IgG2a:IgG1 showed that global IL-4Rα^−/−^ mice had a higher ratio at days 30 (*p* < 0.01) and 56 (*p* < 0.001) p.i. compared with WT and CD4^+^ T cell specific IL-4Rα deficient mice (Figure 6C). Both CD4^+^ T cell specific IL-4Rα deficient mice and wild-type control mice produced comparable amounts of IgE on days 30 and 56 post-infection (Figure 6D). In contrast, no serum IgE was detected for global IL-4Rα^−/−^ mice any day post-infection (Figure 6D). Based on this antibody data, it can be concluded that the inability to signal through the IL-4Rα on CD4^+^ T cells did not prevent a specific type 2 antibody response whereas the inability to class switch in the global IL-4Rα^−/−^ mice resulted in a reduced type 2-antibody response.

### Pan T Cell-Specific IL-4Rα Deficient Mice Remain Comparatively Resistant to VL Compared With Mice Globally Deficient for IL-4/IL-13 Signaling

As CD4^+^ T cell-specific IL-4Rα-deficient mice remain positive for IL-4Rα expression on CD8 T cells, we could not rule out the possibility that IL-4Rα^+^ CD8^+^ T cells in CD4^+^ T cell specific IL-4Rα^−^ deficient mice could have contributed to infection control, especially since CD8^+^ T cells have been implicated in protective immunity to VL (15, 30). Thus, we sought to investigate this further by using pan T cell specific IL-4Rα-deficient mice (iLck^cre^IL-4Rα^−/lox^) which lack IL-4Rα signaling on CD4 T cells as well as CD8 and Foxp3 regulatory T cells (33). iLck^cre^IL-4Rα^−/lox^, IL-4Rα^−/lox^ littermate control and global IL-4Rα^−/−^ mice were infected with *L. donovani* and parasite burdens analyzed at day 30 p.i. in the spleen, liver, and bone-marrow. All groups of animals exhibited similar parasite numbers in the spleen and bone-marrow at day 30 p.i. However, pan T cell IL-4Rα^−/lox^ exhibited disease-control in the liver compared with WT littermate controls (*p* < 0.01) and global IL-4Rα^−/−^ mice (*p* < 0.01, Table 1), similar to but more pronounced than that seen in CD4^+^ T cell-specific IL-4Rα-deficient mice (Figure 1A).

**Table 1:** Parasite burdens of WT (IL-4Rα^−/lox)^, pan T cell IL-4Rα-deficient (iLck^cre^IL-4Rα^−/lox^), and IL-4Rα^−/−^ deficient BALB/c mice in the spleen, liver and bone marrow.

**Strain**	**Mean parasite burden (+ SD)**		
	**Spleen**	**Liver**	**Bone marrow**
IL-4Rα^−/lox^	329 ± 87	2554 ± 334	645 ± 94
iLck^cre^IL-4Rα^−/lox^	285 ± 47	1040 ± 376^**^	494 ± 77
IL-4Rα^−/−^	509 ± 87	2279 ± 376^#^	875 ± 145

*Mice (n = 5/treatment) were infected with L. donovani LV82 and parasite burdens were determined on day 30 post-infection*.

** p < 0.001 compared to WT control, ^**^p < 0.01 compared to WT control,

# *p < 0.01 iLck^cre^IL-4Rα^−/lox^ compared to IL-4Rα^−/−^*.

Altogether, these data indicate that IL-4Rα signaling on T cells transiently exacerbates infection in the liver after infection with *L. donovani*. These results also reinforce the ability of IL-4/IL-13 to exhibit temporal and spatial regulation depending on cell-type and host tissue involved during infection. However, given that IL-4/IL-13 signaling globally is protective in VL, our results clearly demonstrate that the T cell is ultimately not the IL-4Rα responsive target mediating this protection.

## Discussion

While it is well-established that protective immunity against leishmaniasis, whether cutaneous or visceral, relies upon an IL-12-driven type 1 response and IFN-γ production, the contribution of IL-4, IL-13, and signaling via IL-4Rα to the outcome of *Leishmania* infection is very much parasite-species dependent [reviewed (21, 40)]. Numerous studies have identified detrimental roles for IL-4, IL-13, and signaling via IL-4Rα, as well as IL-10, during cutaneous infection with *L. major* and *Leishmania mexicana* complex parasites. In contrast, experimental studies using gene-deficient mice in *L. donovani* infections have indicated that the control of not only primary infection, but also successful chemotherapy and successful vaccination is IL-4, IL-13, and IL-4Rα signaling-dependent (15–17, 32). The absence of these cytokines, or the inability to signal via the IL-4Rα in BALB/c mice, results in a reduced IFN-γ response, severely limited granuloma development, enhanced IL-10 production and disease exacerbation. However, the mode of action of these cytokines continues to remain obscure as IL-4 and IL-13 are pleiotropic cytokines and many cell types are not only able to produce these cytokines but also are responsive to them via IL-4Rα (21). Consequently, the generation of spatial (cell-specific) IL-4Rα^−/−^ mice has provided an invaluable resource to identify the specific role of IL-4/IL-13-responding cells in ongoing immune responses (15, 17, 19). A previous study by us using macrophage/neutrophil specific IL-4Rα-deficient BALB/c mice identified no role, whether protective or detrimental, for IL-4 or IL-13 signaling via these cells on the outcome of primary *L. donovani* infection (16). T cells are the primary source of IFN-γ production in acquired immunity against VL (30), and have been implicated as playing a major role in granuloma formation and resolution of infection in the murine model (39). Therefore, in the present study we utilized CD4^+^ T cell-specific IL-4Rα-deficient (Lck^cre^IL-4Rα^−/lox^) mice (23), and as confirmation iLck^cre^IL-4Rα^−/lox^ mice that lack IL-4Rα on both CD4^+^and CD8^+^ T cells (33) to determine if IL-4Rα signaling via T cells plays any important role in protection. The results reveal that IL-4Rα signaling via the CD4^+^ T cell is not a requirement for successful resolution of *L. donovani* infection, but the inability to signal via IL-4Rα did have a transient protective effect (day 30) on hepatic parasite burdens compared with wild-type counterparts. This hepatic protective effect was also observed in pan-T cell specific BALB/c mice i.e., iLck^cre^IL-4Rα^−/lox^ BALB/c mice, confirming that this early response is mediated by IL-4Rα signaling on T cells. However, later post-infection, CD4^+^ T cell specific IL-4Rα-deficient mice and wild-type controls had equivalent parasite burdens in the spleen, liver and bone marrow as well as a similar hepatic granulomatous response. This is in contrast to global IL-4Rα^−/−^ mice that developed significantly higher parasite burdens and abrogated hepatic granuloma maturation compared with CD4^+^ T cell specific IL-4Rα-deficient and wild-type mice in all tissue sites. Overall these data suggest that host-protection mediated by IL-4/IL-13 globally during *L. donovani* infection is not due to IL-4 acting on CD4^+^ T cell populations.

Earlier studies on *L. donovani*, not only in mice (9), but also humans (10–12), suggested that control of infection was independent of the differential production of type 1 and type 2 cytokines and murine studies indicate that protection is related to increasing the frequency of cytokine-producing cells rather than altering the IFN-γ/IL-4 balance (13). The results presented here confirm that resistance to *L. donovani* and induction of effective granuloma production is dependent on successful generation of an IFN-γ response on both CD4^+^ and CD8^+^ T cells and while this is positively regulated by IL-4/IL-13 signaling on a global scale, it is not strictly dependent on CD4^+^ and CD8^+^ T cells signaling these cytokines. CD4^+^ T cells (26, 39) and CD8^+^ T cells (41) produce IFN-γ, which activates macrophages to produce antimicrobial reactive nitrogen and oxygen intermediates (7), and these are also important in driving granuloma maturation. The observations on comparative T cell IFN-γ expressing cell numbers from this study complement and reinforce our previous observation that global IL-4Rα^−/−^ mice produce significantly lower levels of serum IFN-γ compared with wild-type mice following *L. donovani* infection, and contribute to their abrogated granuloma development (15).

In parallel, although IL-4 is important in proliferation and maintenance of CD4^+^ Th2 cells, it is not required for early production of Th2 cells (42, 43), and therefore the inability to signal via the IL-4Rα did not prevent the induction of antigen-specific Th2 cell response, shown by the similar antigen-specific IgG1 titers in *L. donovani* infected WT and CD4^+^ T cell-specific IL-4Rα-deficient (Lck^cre^IL-4Rα^−/lox^) mice. This may not be unexpected as it has been suggested that antigen-specific Th2 cells is the default position for Th cells (41). The ability to produce antigen-specific IgG1 in the absence of IL-4Rα signaling has been reported in other studies (39, 40).

Recently, a pivotal role for IL-4 and IL-4Rα-responsive B cells in the non-healing response of BALB/c mice infected with *L. major* has been demonstrated (25). Abrogation of IL-4Rα signaling on B cells in BALB/c mice (mb1^cre^IL-4Rα^−/lox^) turned non-healer BALB/c mice into a healer phenotype concomitant with a switch from a predominately Th2 to a Th1 response. Regulatory B cells producing IL-10 have also been associated with non-healing *L. major* infection (44) although apparently not associated with IL-4 responsiveness. However, the role of B cells (as APCs or regulatory cells) and/or antibody in contributing to susceptibility to *Leishmania* infection appears to depend in large part upon the parasite species examined but also probably to a significant extent upon the host. B cells have been shown to play a role in VL, thus B cell-deficient C57BL/6 mice infected with *L. donovani* (45) and BALB/c mice infected with *L. infantum* (46) are relatively resistant to infection. Susceptibility to infection in B cell deficient C57BL/6 mice, unlike BALB/c mice is not dependent on antibody production, although antibodies are associated with protection in infected C57BL/6 mice as they prevent excessive pathology. Marginal zone B cells regulate antigen-specific CD8^+^ T cells responses (47), indicating that B cells may be protective via their ability to present antigen. In *L. infantum*-infected BALB/c mice, neither IL-10 production by B cells nor antigen presentation by B cells is involved in disease exacerbation. However, passive transfer experiments have shown that IgM and IgG, induced by polyclonal B cell activation during infection, promote parasite growth. It is possible that the role of B cells differs in the two mouse strains and is related to their inherent “cure” phenotype (C57BL/6) and “non-cure” phenotype in *L. donovani* (48). B cells have been observed to have cognate interactions with T cells in hepatic granulomas in BALB/c mice (28) and problems with antigen presentation to CD8^+^ T cells have been reported in *L. donovani* (30). There were clear differences in antibody responses between global IL-4Rα^−/−^ mice and the other two strains, with differences in total IgE and antigen-specific IgG1 being the most marked. Production of these antibody classes by B cells is known to be controlled by IL-4/IL-13 and requires IL-4Rα signaling (49). The fact that CD4^+^ T cell specific IL-4Rα^−/−^ mice can still produce IgE indicates that a comparative examination of *L. donovani* disease phenotypes in B cell specific IL-4Rα^−/lox^ BALB/ c mice would be worthwhile.

Both human (12) and murine studies (14, 50) indicate that IL-10, rather than IL-4/IL-13 is the major immunosuppressive cytokine in VL. The source of this IL-10 is not traditional Th2 cells (13) but perhaps a regulatory T cell population (51), and different types of regulatory T cells have been identified in *L. donovani*, including CD4^+^CD25^−^FoxP3^−^ T cells in humans (52), and CD4^+^CD25^−^FoxP3^+^ in mice (53) and humans (54). In addition, numerous non-T cell populations, including DCs (55) and NK cells (56) have been implicated as playing regulatory roles during VL. Thus, *L. donovani* infected IL-10 gene deficient mice demonstrated increased control over visceral infection and enhanced granuloma formation, whilst IL-10 transgenic mice developed a severe progressive disease (50, 57) clearly demonstrating that the presence of IL-10 can prevent efficient granuloma development (50). Indeed, in the present study, global IL-4Rα^−/−^ mice had impaired hepatic granuloma development alongside elevated serum IL-10 levels compared with wild-type control mice and CD4^+^ T cell specific IL-4Rα^−/−^ mice. Interestingly it has previously been shown that IL-4 inhibits IL-10 to promote IL-12 production in DCs in the presence of CpG or LPS (58), or *Cryptococcus neoformans* (59), while IL-13 can induce DC IL-12 production *in vitro* when used to prime DC prior to LPS stimulation (16). IL-4 treatment of BALB/c mice pre-T cell priming has previously been demonstrated to instruct DC to produce IL-12 and facilitate a protective Th1 response against *L. major* (60). In addition, deletion of IL-4Rα on DCs renders BALB/c mice hypersusceptible to *L. major* (22). DC IL-12 production in the early phase of *L. donovani* infection (61–64), in particular, has been identified as directing immune responses influencing granuloma formation during infection. Consequently, these observations, collectively, would clearly point to DCs as very probable targets of the IL-4/IL-13 induced protective response identified from our studies on IL-4^−/−^, IL-13^−/−^, and IL-4Rα^−/−^ mice.

## Conclusion

A significant number of studies from our laboratories have identified protective roles for IL-4, IL-13 and IL-4Rα signaling, not merely during primary infection with *L. donovani* infection (15–17), but also for effective sodium stibogluconate chemotherapy (16, 19). In addition, there is a requirement on IL-4/IL-13 to instruct a protective type 1 response mediated by CD8^+^ T cells in HASPB-1 vaccination against *L. donovani* (65). Given the pleiotropic nature of the IL-4Rα, these IL-4Rα-responsive cells could of course be different populations in different tissue sites and could vary for different type of immune responses e.g., primary infection, response to chemotherapy or successful vaccination. In lieu of this dynamic regulation, as yet our studies have failed to identify the IL-4Rα-expressing cells mediating protection during VL. So far we have ruled out a protective role in VL for IL-4Rα responsiveness on neutrophils and macrophages in primary infection and chemotherapy, and herein T cells during primary infection. Interestingly, the primary source of IL-4 in HASPB-1 vaccination studies was defined as a CD11b^+^CD11c^lo^ phagocyte (65) and as this source is clearly not macrophages/neutrophils (16), alternate phagocytes expressing CD11b may regulate host immunity to VL. A candidate phagocyte for this role could be DCs given the “IL-4 instruction theory,” defined as the ability of early IL-4, signaling via the IL-4Rα on DCs, to instruct early IL-12 production to promote Th1-responses (22, 60). We are currently investigating the exact mechanism to explain how this works as well as generating a variety of additional IL-4Rα cell specific knockouts to allow further dissection of the immunological mechanisms responsible for IL-4Rα mediated protection against VL. Ultimately identifying the IL-4Rα responsive host cells mediating protection will have significant implications in the rational design of new host-directed therapeutic strategies.

## Data Availability

The datasets generated for this study are available on request to the corresponding author.

## Author Contributions

JA, FB, and PK: conceptualization. EM, KC, TM, and RH: methodology and investigation. EM, KC, JA, and RH: writing, review, and editing of manuscript. FB: cell-specific gene-deficient mice.

### Conflict of Interest Statement

The authors declare that the research was conducted in the absence of any commercial or financial relationships that could be construed as a potential conflict of interest.

## References

[1] Sundar S Singh A. Chemotherapeutics of visceral leishmaniasis: present and future developments. Parasitology. (2018) 145:481–9. 10.1017/S0031182017002116 29215329 PMC5984184

[2] Hotez PJ. The global fight to develop antipoverty vaccines in the anti-vaccine era. Hum Vaccin Immunother. (2018) 14:2128–31. 10.1080/21645515.2018.1430542 29393710 PMC6183138

[3] Zumla A Rao M Wallis RS Kaufmann SH Rustomjee R Mwaba P. Host-directed therapies for infectious diseases: current status, recent progress, and future prospects. Lancet Infect Dis. (2016) 16:e47–63. 10.1016/S1473-3099(16)00078-5 27036359 PMC7164794

[4] Hayes AJ Rane S Scales HE Meehan GR Benson RA Maroof A. Spatiotemporal modeling of the key migratory events during the initiation of adaptive immunity. Front Immunol. (2019) 10:598. 10.3389/fimmu.2019.00598 31024523 PMC6460458

[5] Engwerda CR Murphy ML Cotterell SE Smelt SC Kaye PM. Neutralization of IL-12 demonstrates the existence of discrete organ- specific phases in the control of *Leishmania donovani*. Eur J Immunol. (1998) 28:669–80. 9521077 10.1002/(SICI)1521-4141(199802)28:02<669::AID-IMMU669>3.0.CO;2-N

[6] Murray HW Hariprashad J Coffman RL. Behavior of visceral *Leishmania donovani* in an experimentally induced T helper cell 2 (Th2)-associated response model. J Exp Med. (1997) 185:867–74. 10.1084/jem.185.5.867 9120392 PMC2196164

[7] Murray HW Nathan CF. Macrophage microbicidal mechanisms *in vivo*: reactive nitrogen versus oxygen intermediates in the killing of intracellular visceral *Leishmania donovani*. J Exp Med. (1999) 189:741–6. 10.1084/jem.189.4.741 9989990 PMC2192937

[8] Siewe N Yakubu AA Satoskar AR Friedman A. Granuloma formation in leishmaniasis: a mathematical model. J Theor Biol. (2017) 412:48–60. 10.1016/j.jtbi.2016.10.004 27769685

[9] Kaye PM Curry AJ Blackwell JM. Differential production of Th1- and Th2-derived cytokines does not determine the genetically controlled or vaccine-induced rate of cure in murine visceral leishmaniasis. J Immunol. (1991) 146:2763–70. 1901883

[10] Karp CL el-Safi SH Wynn TA Satti MM Kordofani AM Hashim FA. *in vivo* cytokine profiles in patients with kala-azar. Marked elevation of both interleukin-10 and interferon-gamma. J Clin Invest. (1993) 91:1644–8. 10.1172/JCI116372 8097208 PMC288142

[11] Kemp K. Cytokine-producing T cell subsets in human leishmaniasis. Arch Immunol Ther Exp. (2000) 48:173–6. 10912621

[12] Kemp M Kurtzhals JA Bendtzen K Poulsen LK Hansen MB Koech DK. *Leishmania donovani*-reactive Th1- and Th2-like T-cell clones from individuals who have recovered from visceral leishmaniasis. Infect Immun. (1993) 61:1069–73. 8432588 10.1128/iai.61.3.1069-1073.1993 PMC302840

[13] Murphy ML Cotterell SE Gorak PM Engwerda CR Kaye PM. Blockade of CTLA-4 enhances host resistance to the intracellular pathogen, *Leishmania donovani*. J Immunol. (1998) 161:4153–60. 9780188

[14] Murphy ML Wille U Villegas EN Hunter CA Farrell JP. IL-10 mediates susceptibility to *Leishmania donovani* infection. Eur J Immunol. (2001) 31:2848–56. 10.1002/1521-4141(2001010)31:10<2848::AID-IMMU2848>3.0.CO;2-T 11592059

[15] Stäger S Alexander J Carter KC Brombacher F Kaye PM. Both interleukin-4 (IL-4) and IL-4 receptor alpha signaling contribute to the development of hepatic granulomas with optimal antileishmanial activity. Infect Immun. (2003) 71:4804–7. 10.1128/IAI.71.8.4804-4807.2003 12874364 PMC166035

[16] McFarlane E Carter KC McKenzie AN Kaye PM Brombacher F Alexander J. Endogenous IL-13 plays a crucial role in liver granuloma maturation during *Leishmania donovani* infection, independent of IL-4Ralpha-responsive macrophages and neutrophils. J Infect Dis. (2011) 204:36–43. 10.1093/infdis/jir080 21628656 PMC3105032

[17] Satoskar A Bluethmann H Alexander J Disruption of the murine interleukin-4 gene inhibits disease progression during *Leishmania mexicana* infection but does not increase control of *Leishmania donovani* infection. Infect Immun. (1995) 63:4894–9. 7591152 10.1128/iai.63.12.4894-4899.1995 PMC173701

[18] Murray HW Montelibano C Peterson R Sypek JP. Interleukin-12 regulates the response to chemotherapy in experimental visceral Leishmaniasis. J Infect Dis. (2000) 182:1497–502. 10.1086/315890 11023473

[19] Alexander J Carter KC Al-Fasi N Satoskar A Brombacher F. Endogenous IL-4 is necessary for effective drug therapy against visceral leishmaniasis. Eur J Immunol. (2000) 30:2935–43. 10.1002/1521-4141(200010)30:10<2935::AID-IMMU2935>3.0.CO;2-Q 11069076

[20] Roy S Mukhopadhyay D Mukherjee S Moulik S Chatterji S Brahme N. An IL-10 dominant polarization of monocytes is a feature of Indian Visceral leishmaniasis. Parasite Immunol. (2018) 40:e12535. 10.1111/pim.12535 29745990

[21] Hurdayal R Brombacher F. Interleukin-4 receptor alpha: from innate to adaptive immunity in murine models of cutaneous leishmaniasis. Front Immunol. (2017) 8:1354. 10.3389/fimmu.2017.01354 29176972 PMC5686050

[22] Hurdayal R Nieuwenhuizen NE Revaz-Breton M Smith L Hoving JC Parihar SP. Deletion of IL-4 receptor alpha on dendritic cells renders BALB/c mice hypersusceptible to Leishmania major infection. PLoS Pathog. (2013) 9:e1003699. 10.1371/journal.ppat.1003699 24204259 PMC3812013

[23] Radwanska M Cutler AJ Hoving JC Magez S Holscher C Bohms A. Deletion of IL-4Ralpha on CD4 T cells renders BALB/c mice resistant to leishmania major infection. PLoS Pathog. (2007) 3:e68. 10.1371/journal.ppat.0030068 17500591 PMC1867380

[24] Hölscher C Arendse B Schwegmann A Myburgh E Brombacher F. Impairment of alternative macrophage activation delays cutaneous leishmaniasis in nonhealing BALB/c mice. J Immunol. (2006) 176:1115–21. 10.4049/jimmunol.176.2.1115 16394000

[25] Hurdayal R Ndlovu HH Revaz-Breton M Parihar SP Nono JK Govender M. IL-4-producing B cells regulate T helper cell dichotomy in type 1- and type 2-controlled diseases. Proc Natl Acad Sci USA. (2017)114:E8430–9. 10.1073/pnas.1708125114 28916732 PMC5635893

[26] Bunn PT Stanley AC de Labastida Rivera F Mulherin A Sheel M Alexander CE. Tissue requirements for establishing long-term CD4+ T cell-mediated immunity following *Leishmania donovani* infection. J Immunol. (2014) 192:3709–18. 10.4049/jimmunol.1300768 24634490

[27] Owens BM Beattie L Moore JW Brown N Mann JL Dalton JE. IL-10-producing Th1 cells and disease progression are regulated by distinct CD11c(+) cell populations during visceral leishmaniasis. PLoS Pathog. (2012) 8:e1002827. 10.1371/journal.ppat.1002827 22911108 PMC3406093

[28] Moore JW Beattie L Dalton JE Owens BM Maroof A Coles MC. B cell: T cell interactions occur within hepatic granulomas during experimental visceral leishmaniasis. PLoS ONE. (2012) 7:e34143. 10.1371/journal.pone.0034143 22479545 PMC3316612

[29] Moore JW Moyo D Beattie L Andrews PS Timmis J Kaye PM. Functional complexity of the leishmania granuloma and the potential of *in silico* modeling. Front Immunol. (2013) 4:35. 10.3389/fimmu.2013.00035 23423646 PMC3573688

[30] Stäger S Rafati S CD8(+) T cells in leishmania infections: friends or foes? Front Immunol. (2012) 3:5 10.3389/fimmu.2012.00005 22566891 PMC3342007

[31] Murray HW Lu CM Brooks EB Fichtl RE DeVecchio JL Heinzel FP Modulation of T-cell costimulation as immunotherapy or immunochemotherapy in experimental visceral leishmaniasis. Infect Immun. (2003) 71:6453–62. 10.1128/IAI.71.11.6453-6462.2003 14573667 PMC219611

[32] Stäger S Smith DF Kaye PM. Immunization with a recombinant stage-regulated surface protein from *Leishmania donovani* induces protection against visceral leishmaniasis. J Immunol. (2000) 165:7064–71. 10.4049/jimmunol.165.12.7064 11120835

[33] Dewals B Hoving JC Leeto M Marillier RG Govender U Cutler AJ. IL-4Ralpha responsiveness of non-CD4 T cells contributes to resistance in schistosoma mansoni infection in pan-T cell-specific IL-4Ralpha-deficient mice. Am J Pathol. (2009) 175:706–16. 10.2353/ajpath.2009.090137 19628763 PMC2716945

[34] Herbert DR Hölscher C Mohrs M Arendse B Schwegmann A Radwanska M. Alternative macrophage activation is essential for survival during schistosomiasis and downmodulates T helper 1 responses and immunopathology. Immunity. (2004) 20:623–35. 10.1016/S1074-7613(04)00107-4 15142530

[35] Mohrs M Ledermann B Köhler G Dorfmüller A Gessner A Brombacher F. Differences between IL-4- and IL-4 receptor alpha-deficient mice in chronic leishmaniasis reveal a protective role for IL-13 receptor signaling. J Immunol. (1999) 162:7302–8. 10358179

[36] Gu H Marth JD Orban PC Mossmann H Rajewsky K. Deletion of a DNA polymerase beta gene segment in T cells using cell type-specific gene targeting. Science. (1994) 265:103–6. 10.1126/science.8016642 8016642

[37] Garvin AM Abraham KM Forbush KA Farr AG Davison BL Perlmutter RM. Disruption of thymocyte development and lymphomagenesis induced by SV40 T-antigen. Int Immunol. (1990) 2:173–80. 10.1093/intimm/2.2.173 1965144

[38] Carter KC Sundar S Spickett C Pereira OC Mullen AB. The *in vivo* susceptibility of *Leishmania donovani* to sodium stibogluconate is drug specific and can be reversed by inhibiting glutathione biosynthesis. Antimicrob Agents Chemother. (2003) 47:1529–35. 10.1128/AAC.47.5.1529-1535.2003 12709318 PMC153333

[39] Murray HW. Tissue granuloma structure-function in experimental visceral leishmaniasis. Int J Exp Pathol. (2001) 82:249–67. 10.1046/j.1365-2613.2001.00199.x 11703536 PMC2517779

[40] Alexander J Brombacher F. T helper1/t helper2 cells and resistance/susceptibility to leishmania infection: is this paradigm still relevant? Front Immunol. (2012) 3:80. 10.3389/fimmu.2012.00080 22566961 PMC3342373

[41] Stern JJ Oca MJ Rubin BY Anderson SL Murray HW. Role of L3T4+ and LyT-2+ cells in experimental visceral leishmaniasis. J Immunol. (1988) 140:3971–7. 3131421

[42] Brewer JM Conacher M Hunter CA Mohrs M Brombacher F Alexander J Aluminium hydroxide adjuvant initiates strong antigen-specific Th2 responses in the absence of IL-4- or IL-13-mediated signaling. J Immunol. (1999) 163:6448–54. 10586035

[43] Brewer JM Conacher M Satoskar A Bluethmann H Alexander J. In interleukin-4-deficient mice, alum not only generates T helper 1 responses equivalent to freund's complete adjuvant, but continues to induce T helper 2 cytokine production. Eur J Immunol. (1996) 26:2062–6. 10.1002/eji.1830260915 8814247

[44] Ronet C Hauyon-La Torre Y Revaz-Breton M Mastelic B Tacchini-Cottier F Louis J. Regulatory B cells shape the development of Th2 immune responses in BALB/c mice infected with leishmania major through IL-10 production. J Immunol. (2010) 184:886–94. 10.4049/jimmunol.0901114 19966209

[45] Smelt SC Cotterell SE Engwerda CR Kaye PM. B cell-deficient mice are highly resistant to *Leishmania donovani* infection, but develop neutrophil-mediated tissue pathology. J Immunol. (2000) 164:3681–8. 10.4049/jimmunol.164.7.3681 10725726

[46] Deak E Jayakumar A Cho KW Goldsmith-Pestana K Dondji B Lambris JD. Murine visceral leishmaniasis: IgM and polyclonal B-cell activation lead to disease exacerbation. Eur J Immunol. (2010) 40:1355–68. 10.1002/eji.200939455 20213734 PMC2944234

[47] Bankoti R Gupta K Levchenko A Stäger S. Marginal zone B cells regulate antigen-specific T cell responses during infection. J Immunol. (2012) 188:3961–71. 10.4049/jimmunol.1102880 22412197

[48] Blackwell J Freeman J Bradley D. Influence of H-2 complex on acquired resistance to *Leishmania donovani* infection in mice. Nature. (1980) 283:72–4. 10.1038/283072a0 6765984

[49] Noben-Trauth N Shultz LD Brombacher F Urban JF Gu H Paul WE. An interleukin 4 (IL-4)-independent pathway for CD4+ T cell IL-4 production is revealed in IL-4 receptor-deficient mice. Proc Natl Acad Sci USA. (1997) 94:10838–43. 10.1073/pnas.94.20.10838 9380721 PMC23501

[50] Murray HW Lu CM Mauze S Freeman S Moreira AL Kaplan G. Interleukin-10 (IL-10) in experimental visceral leishmaniasis and IL-10 receptor blockade as immunotherapy. Infect Immun. (2002) 70:6284–93. 10.1128/IAI.70.11.6284-6293.2002 12379707 PMC130311

[51] Bodas M Jain N Awasthi A Martin S Penke Loka RK Dandekar D. Inhibition of IL-2 induced IL-10 production as a principle of phase-specific immunotherapy. J Immunol. (2006) 177:4636–43. 10.4049/jimmunol.177.7.4636 16982902

[52] Nylén S Maurya R Eidsmo L Manandhar KD Sundar S Sacks D. Splenic accumulation of IL-10 mRNA in T cells distinct from CD4+CD25+ (Foxp3) regulatory T cells in human visceral leishmaniasis. J Exp Med. (2007) 204:805–17. 10.1084/jem.20061141 17389235 PMC2118563

[53] Tiwananthagorn S Iwabuchi K Ato M Sakurai T Kato H Katakura K. Involvement of CD4(+) Foxp3(+) regulatory T cells in persistence of *Leishmania donovani* in the liver of alymphoplastic aly/aly mice. PLoS Negl Trop Dis. (2012) 6:e1798. 10.1371/journal.pntd.0001798 22928057 PMC3424244

[54] Bhattacharya P Ghosh S Ejazi SA Rahaman M Pandey K Ravi Das VN. Induction of IL-10 and TGFbeta from CD4+CD25+FoxP3+ T cells correlates with parasite load in Indian Kala-azar patients infected with *Leishmania donovani*. PLoS Negl Trop Dis. (2016) 10:e0004422. 10.1371/journal.pntd.0004422 26829554 PMC4735109

[55] Svensson M Maroof A Ato M Kaye PM. Stromal cells direct local differentiation of regulatory dendritic cells. Immunity. (2004) 21:805–16. 10.1016/j.immuni.2004.10.012 15589169

[56] Maroof A Beattie L Zubairi S Svensson M Stager S Kaye PM. Posttranscriptional regulation of II10 gene expression allows natural killer cells to express immunoregulatory function. Immunity. (2008) 29:295–305. 10.1016/j.immuni.2008.06.012 18701085 PMC2656759

[57] Murray HW Flanders KC Donaldson DD Sypek JP Gotwals PJ Liu J. Antagonizing deactivating cytokines to enhance host defense and chemotherapy in experimental visceral leishmaniasis. Infect Immun. (2005) 73:3903–11. 10.1128/IAI.73.7.3903-3911.2005 15972476 PMC1168607

[58] Yao Y Li W Kaplan MH Chang CH. Interleukin (IL)-4 inhibits IL-10 to promote IL-12 production by dendritic cells. J Exp Med. (2005) 201:1899–903. 10.1084/jem.20050324 15967820 PMC2212025

[59] Grahnert A Richter T Piehler D Eschke M Schulze B Müller U. IL-4 receptor-alpha-dependent control of *Cryptococcus neoformans* in the early phase of pulmonary infection. PLoS ONE. (2014) 9:e87341. 10.1371/journal.pone.0087341 24475277 PMC3903725

[60] Biedermann T Zimmermann S Himmelrich H Gumy A Egeter O Sakrauski AK. IL-4 instructs TH1 responses and resistance to Leishmania major in susceptible BALB/c mice. Nat Immunol. (2001) 2:1054–60. 10.1038/ni725 11600887

[61] Engwerda CR Murphy ML Cotterell SE Smelt SC Kaye PM. Neutralization of IL-12 demonstrates the existence of discrete organ-specific phases in the control of *Leishmania donovani*. Eur J Immunol. (1998) 28:669–80. 9521077 10.1002/(SICI)1521-4141(199802)28:02<669::AID-IMMU669>3.0.CO;2-N

[62] Gorak PM Engwerda CR Kaye PM Dendritic cells, but not macrophages, produce IL-12 immediately following *Leishmania donovani* infection. Eur J Immunol. (1998) 28:687–95. 9521079 10.1002/(SICI)1521-4141(199802)28:02<687::AID-IMMU687>3.0.CO;2-N

[63] Murray HW. Endogenous interleukin-12 regulates acquired resistance in experimental visceral leishmaniasis. J Infect Dis. (1997) 175:1477–9. 10.1086/516482 9180189

[64] Stanley AC Dalton JE Rossotti SH MacDonald KP Zhou Y Rivera F. VCAM-1 and VLA-4 modulate dendritic cell IL-12p40 production in experimental visceral leishmaniasis. PLoS Pathog. (2008) 4:e1000158. 10.1371/journal.ppat.1000158 18802456 PMC2528938

[65] Stäger S Alexander J Kirby AC Botto M Rooijen NV Smith DF. Natural antibodies and complement are endogenous adjuvants for vaccine-induced CD8+ T-cell responses. Nat Med. (2003) 9:1287–92. 10.1038/nm933 14502281

