# Supplementary material for: IL-4 Mediated Resistance of BALB/c Mice to Visceral Leishmaniasis Is Independent of IL-4Rα Signaling via T Cells
Source: Front Immunol. 2019 Aug 16;10:1957. doi: 10.3389/fimmu.2019.01957 (PMC6707061; doi:10.3389/fimmu.2019.01957)

IL-4 mediated resistance of BALB/c mice to visceral leishmaniasis is independent of IL-4Rα signalling via T cells'.

SUPPLEMENTARY INFORMATION

Page

7 **Table S1:** *L. donovani* parasite burdens in the spleen, liver and bone marrow of $CD4^+$ T cell-specific IL-4Rα-deficient (Lck^{cre}IL-4R$\alpha^{-/lox}$) mice, wild-type littermate control (IL-4R$\alpha^{-/lox}$) and global IL-4R$\alpha^{-/-}$ BALB/c mice at days 14 or 15 post-infection.

7 **Table S2:** Granuloma maturation in *L. donovani* infected $CD4^+$ T cell-specific IL-4Rα-deficient (Lck^{cre}IL-4R$\alpha^{-/lox}$) mice, wild-type littermate control (IL-4R$\alpha^{-/lox}$) and global IL-4R$\alpha^{-/-}$ BALB/c mice at day 14 and 15 post-infection.

8 **Figure S1:** Representative photomicrographs of the hepatic granuloma response in an *L. donovani* infected wild-type (IL-4R$\alpha^{-/lox}$), $CD4^+$ T cell-specific IL-4Rα-deficient (Lck^{cre}IL-4R$\alpha^{-/lox}$), and global IL-4R$\alpha^{-/-}$ mice at days 15 post-infection.

Table S1: *L. donovani* parasite burdens in the spleen, liver and bone marrow of $CD4^+$ T cell-specific IL-4Rα-deficient ($Lck^{cre}IL\text{-}4R\alpha^{-/lox}$), wild-type littermate control ($IL\text{-}4R\alpha^{-/lox}$) and global $IL\text{-}4R\alpha^{-/-}$ BALB/c mice at different times post-infection. Mice were infected with *L. donovani* on day 0 and parasite burdens were determined on day 14 or 15 in separate experiments.

**Treatment**	**Mean parasite burden ± SE**		
	**Spleen**	**Liver**	**Bone marrow**
**Day 14**			
WT ($IL\text{-}4R\alpha^{-/lox}$)	194 ± 16	4144 ± 256	1038 ± 31
$CD4^+$ T cell-specific IL-4Rα-deficient ($Lck^{cre}IL\text{-}4R\alpha^{-/lox}$)	223 ± 45	3597 ± 560	947± 108
Global $IL\text{-}4R\alpha^{-/-}$	214± 31	4223 ± 337	788 ± 181
**Day 15**			
WT ($IL\text{-}4R\alpha^{-/lox}$)	91 ± 16	2349 ± 192	387 ± 106
$CD4^+$ T cell-specific IL-4Rα-deficient ($Lck^{cre}IL\text{-}4R\alpha^{-/lox}$)	143 ± 19	2312 ± 192	528 ± 99

Table S2: Granuloma maturation in *L. donovani* infected $CD4^+$ T cell-specific IL-4Rα-deficient ($Lck^{cre}IL\text{-}4R\alpha^{-/lox}$), wild-type littermate control ($IL\text{-}4R\alpha^{-/lox}$) and global $IL\text{-}4R\alpha^{-/-}$ BALB/c mice at day 14 and 15 post-infection in separate experiments. ND – not determined.

**Granuloma stage**	**Strain**		
	**WT control ($IL\text{-}4R\alpha^{-/lox}$)**	**$CD4^+$ T cell-specific IL-4Rα-deficient ($Lck^{cre}IL\text{-}4R\alpha^{-/lox}$)**	**Global $IL\text{-}4R\alpha^{-/-}$**
**Day 14**			
Sterile	1.5 ± 1.1	0.4 ± 0.7	0.6 ± 1.1
Mature	6.8 ± 1.1	3.1 ± 1.7	2.7 ± 2.7
Immature	29.6 ± 7.1	22.7 ± 6.4	17.4 ± 5.6
Kupffer cells	61.9 ± 6.7	73.3 ± 7.9	67.2 ± 8.3
**Day 15**			
Sterile	2.00 ± 0.8	1.03 ± 0.9	ND
Mature	8 ± 2.5	5.83 ± 0.9	ND
Immature	28.18 ± 3.3	30.30 ± 3.6	ND
Kupffer cells	59.32 ± 9.0	62.5 ± 3.3	ND

Fig. S1: Representative photomicrographs of the hepatic granuloma response in *L. donovani* infected wild-type (IL-4R$\alpha^{-/lox}$), CD4^{+} T cell-specific IL-4Rα-deficient (Lck^{cre}IL-4R$\alpha^{-/lox}$) and global IL-4R$\alpha^{-/-}$ mice at day 15 post-infection. Immature granulomas and amastigotes within the Kupffer cells at Day 15 are denoted by (arrows).

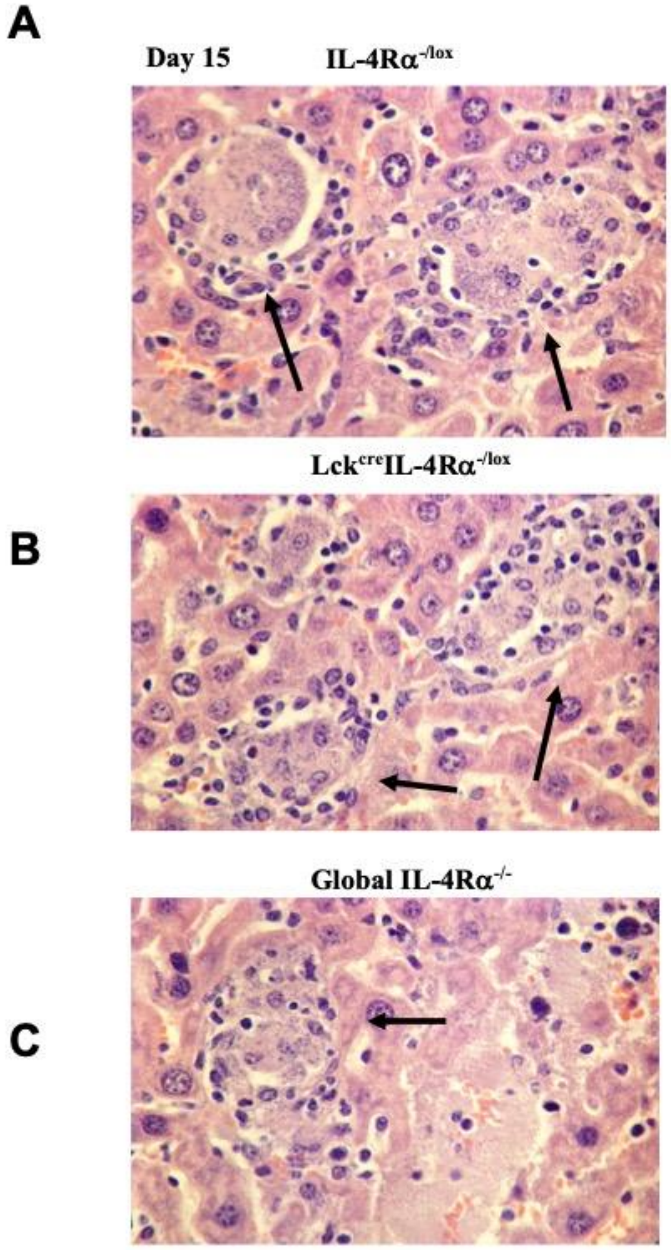

Supplement: Supplementary file 1 [file Data_Sheet_1.pdf]
